# The application of anticoagulant therapy to sepsis

**DOI:** 10.1186/s40560-017-0230-3

**Published:** 2017-05-30

**Authors:** Jecko Thachil, Toshiaki Iba

**Affiliations:** 1 Department of Haematology, Manchester Royal Infirmary, Manchester, UK; 2 Department of Emergency and Disaster Medicine, Graduate School of Medicine, Juntendo University, 2-1-1 Hongo Bunkyo-ku, Tokyo, 113-8421 Japan

**Keywords:** Sepsis, Anticoagulants, Disseminated intravascular coagulation

## Abstract

Since the withdrawal of recombinant-activated protein C, the anticoagulant therapy for sepsis was taken no notice. For instance, the international sepsis guidelines did not share a space for this therapy. However, we can see the signs of change recently. The Surviving Sepsis Campaign Guidelines 2016 introduced the development of recombinant thrombomodulin, and the *European Society of Intensive Care Medicine* released comments on this subject. However, since small but important discrepancy was recognized between the standpoints of Japan (more aggressive) and other countries (rather conservative), we intend to discuss on this point.

## Main text

Recently, the *Intensive Care Medicine* published a pro and con debate entitled ‘Should all patients with sepsis receive anticoagulation?’ [1, 2]. We read the above articles with surprise since the anticoagulant therapies had been almost ignored in the countries other than Japan. Though these editorials were concise, they contain many important messages. Thus, we would like to introduce the essence of those articles and try to make some comments on them.

Septic coagulopathy represented by disseminated intravascular coagulation (DIC) is Janus-faced. It is a part of the frontliners of the host defence against infection but induces organ dysfunction at the same time [3]. Meziani et al. [1] mentioned that the coagulation disorder is an almost universal event in sepsis, and thus, anticoagulation may have favourable effects from the standpoint of pros; however, we think we have to remind that not all patients can get benefits from the anticoagulant therapy. Numbers of randomized controlled trials (RCT) failed to show the benefit of multiple agents. From the standpoint of cons, van der Poll et al. [2] asserted that the universal administration of anticoagulants cannot be recommenced because of the increased risk of bleeding and the possibility of hampering coagulation-assisted clearance of pathogens. Well, we agree on both opinions and suppose that the selection of the right target is the fundamental condition for the success in the treatment. Indeed, the recent studies repeatedly reported the beneficial effect was seen only in the patients with severe coagulation disorder [4]. In addition, the subgroup analyses in the KyberSept and PROWESS trials revealed the trend towards greater risk reduction in mortality in the patients with DIC than in the patients without [5, 6]. Therefore, we think the stratification to identify the most suitable patient population is essential for the success in the anticoagulant therapies. More detailed analysis is required to find the adequate timing, dose, and duration for each anticoagulant. For that purpose, we insist the need for more active investigation; performing not only the RCTs but also the other styles of clinical studies should be carried out. Regarding the international difference, we realized the prevalence of anticoagulation is more dominant in Japan since the diagnosis of DIC has been a part of the standard care for sepsis management, whereas the other countries are more conservative because the measurements of the coagulation markers are not the routine work. This difference should influence the result of the anticoagulant therapy.

The current obstacle is the lack of the appropriate diagnostic tool. There are several popular diagnostic criteria for DIC, but none of them were specially designed for sepsis-associated DIC. Sepsis-induced coagulopathy (SIC) is uniquely characterized by coagulation activation with over-suppression of fibrinolysis and a high incidence of organ dysfunction [7]. Thus, we need to establish proper diagnostic criteria for SIC by using the data from homogenous group of patients with the same basic pathophysiology and clinical characteristics. For example, we have ever introduced the diagnostic criteria that include the baseline antithrombin activity for the application of antithrombin substitution [8]. With regard to the use of thrombomodulin, a large-scale randomized controlled trial (RCT) is in progress, a phase III study for recombinant human soluble thrombomodulin [9]. This RCT targets on the subset of sepsis patients with organ dysfunction and prothrombin time INR of more than 1.4. However, whether this category is adequate has not been validated yet. For the adequate target of recombinant thrombomodulin, Yamakawa et al. [10] revealed a significant correlation between effect size and baseline severity, and Yoshimura et al. [11] reported that the effect was most remarkable when the baseline APACHE II score was between 24 and 29. Eventually, we propose to include the indicator of organ dysfunction for the future design of SIC. Though we still do not know what are the ideal indicators, serum lactate level, damage-associated molecular markers, thrombelastography, thrombin generation test, and clot waveform analysis are the possible candidates. In the recent editorial in *Intensive Care Medicine*, Levi M. et al. introduced the usefulness of measuring microparticles, and clot strength and elasticity as another options [12].

In contrast to the international ‘Surviving Sepsis Campaign Guidelines 2016 (SSCG)’, the latest ‘Japanese Clinical Practice Guidelines for Management of Sepsis and Septic Shock (J-SSCG) [13]’ recommend the supplementation of antithrombin for sepsis patients with DIC. The background of the discrepancy is explained as follows: though the largest RCT named KyberSept failed to show the survival benefit in the group treated with antithrombin, the subgroup analysis found the positive signal from the treatment [5]. SSCG adopted the result of KyberSept, and J-SSCG adopted it from the subgroup analysis. In contrast to the positive evaluation for antithrombin, the recommendation for recombinant thrombomodulin was postponed in both guidelines since the sufficient data has not been obtained yet.

During the course of setting the guidelines, the cost of anticoagulation was the subject of discussion. The anticoagulant therapy with antithrombin for 3 days costs approximately US$2000, and 6 days treatment with recombinant thrombomodulin costs almost US$4000. These medical expenses are the common prices when patients are administered antithrombin or thrombomodulin for DIC treatment in Japan, and the cost-effectiveness is an unsolved issue of these therapies. For more convincing evaluation, advanced analyses such as the comparisons of ICU-free days, hospitalization-free days, and total cost should be performed. In addition, the risk of bleeding is the other issue. It may be permissive [14], but still not ignorable especially in the patients who underwent surgical interventions. The risk of bleeding was reported to be high, when the high dose of antithrombin was applied. Indeed, in the KyberSept trial, total of 30,000 IU (loading dose of 6000 IU, followed by a continuous infusion of 6000 IU/day for 4 days) was administered and the bleeding incidence was over 20% [15]. In contrast, the incidence was around 5% when the supplemental dose (1500 IU/day for 3 days) was normally administered for the treatment of DIC in Japan. As for recombinant thrombomodulin, Eguchi et al. [16] reported that serious bleeding was recognized in 121 cases out of 1787 sepsis-induced DIC patients treated with recombinant thrombomodulin (6.8%) in their post-marketing survey. In contrast, the phase 2b study performed outside Japan reported the serious bleeding was recognized in 19 out of 370 patients (5.1%) [17].

In summary, the observation that not one but various anticoagulants are effective suggests the effectiveness of anticoagulant therapy, and the result that the efficacy was recognized only in the patients with severe coagulation disorder further supports this idea. Though high cost and risk of bleeding are possible disadvantages, we think the anticoagulation may be one of the most promising treatments for septic coagulopathy.

## Abbreviations

DIC: Disseminated intravascular coagulation
RCT: Randomized controlled trial
SIC: Sepsis-induced coagulopathy
